# Diagnosing SARS-CoV-2 vaccination associated rhombencephalitis requires comprehensive work-up and exclusion of differentials

**DOI:** 10.1186/s42466-022-00178-9

**Published:** 2022-03-21

**Authors:** Josef Finsterer, Fulvio A. Scorza, Carla A. Scorza, Ana C. Fiorini

**Affiliations:** 1 Neurology and Neurophysiology Center, Postfach 20, 1180 Vienna, Austria; 2 Disciplina de Neurociência, Universidade Federal de São Paulo/Escola Paulista de Medicina (UNIFESP/EPM), São Paulo, Brazil

**Keywords:** SARS-CoV-2, COVID-19, Vaccination, Immune-mediated, Guillain–Barre syndrome, Encephalitis

## Abstract

In this letter we raise several concerns regarding the interesting article by Walter and Krämer about rhomb-encephalitis as a complication two months after the vaccination with an mRNA-based SARS-CoV_2 vaccine. The causal link between the vaccination and encephalitis remained unproven, a SARS-CoV-2 infection, Bickerstaff encephalitis were not excluded, the MRI rather suggests brainstem-encephlaitis than pure rhomb-encephalitis, and the cerebro-spinal fluid was not investigated for cytokines or glial markers. Neurologists are called to make all available effort to convincingly evaluate the etiology and the pathophysiological background of an undetermined condition.

To the Editor

With interest we read the article by Walter and Krämer about the case of a 30 years old neurologist who was diagnosed with rhomb-encephalitis about two months after the second jab of a SARS-CoV-2 vaccination with an mRNA-based vaccine (mRNA-1273, Moderna) [1]. A causal link between the vaccination and the neurological condition was established [1]. The patient benefited from steroids and achieved almost complete recovery [1]. The study is appealing but raises comments and concerns.

The main limitation of the study is that a causal relation between the vaccination and the neurological abnormalities was not convincingly established. A strong argument against a causal relation is the long latency of about two months between the vaccination and the onset of the neurological deficits. No pathophysiological explanation was provided why two months after a SARS-CoV-2 vaccination all of a sudden a severe neurological condition occurred.

A further limitation is that the results of the SARS-CoV-2 tests on admission and throughout hospitalisation were not provided. As encephalitis is a known complication of SARS-CoV-2 infections [2], and as it is known that SARS-CoV-2 infections occur despite full immunisation [3], it is conceivable that the patient experienced a SARS-CoV-2 infection with a neurological manifestation.

Furthermore, we are not convinced that the condition represents indeed rhomb-encephalitis. An argument against encephalitis is that the lesions shown on MRI did not enhance [1]. A further limitation is that no follow-up MRI was provided to document that the lesions had disappeared with resolution of the symptoms after treatment. Since there was also a lesion in the midbrain the condition should be rather termed brainstem encephalitis instead of rhomb-encephalitis. The lesions seen on MRI could also represent Bickerstaff encephalitis, a subtype of Guillain–Barre syndrome (GBS). As GBS has been reported as a complication of a SARS-CoV-2 vaccination in almost 400 cases as per the end of September 2021 [4] and immune encephalitis only rarely, it cannot be excluded that the condition represents Bickerstaff encephalitis rather than rhomb-encephalitis.

Another limitation is that the cerebro-spinal fluid (CSF) was not investigated for SARS-CoV-2 or immunological parameters, such as the cytokines interleukin (IL) 1a, IL-6 or IL-8, TNF-alpha, beta-2 microglobulin, and glial markers, which have been reported elevated in the CSF of SARS-CoV-2 related encephalitis [5].

We should also be informed about the exact latency between the Moderna jab and the clinical onset of symptoms.

Overall, the interesting study has several limitations which challenge the results and their interpretation. We agree that neurologists should stay vigilant as not to overlook neurological side effects of SARS-CoV-2 vaccinations. However, neurologists are also called to make all available effort to convincingly evaluate the etiology and the pathophysiological background of an undetermined condition.
